# Supplementary material for: Exposure to Endocrine Disruptors (Di(2-Ethylhexyl)phthalate (DEHP) and Bisphenol A (BPA)) in Women from Different Residing Areas in Italy: Data from the LIFE PERSUADED Project
Source: Int J Mol Sci. 2022 Dec 16;23(24):16012. doi: 10.3390/ijms232416012 (PMC9783390; doi:10.3390/ijms232416012)
Supplement: Supplementary file 1 [file ijms-23-16012-s001.zip › ijms-2014939-supplementary.pdf]

Supplementary Tables for the *Article*

Exposure to endocrine disruptors (Di(2-ethylhexyl)phthalate (DEHP) and bisphenol A (BPA)) in women from different residing areas in Italy: data from the LIFE PERSUADED project

Fabrizia Carli [1], Sabrina Tait [2], Luca Busani [2], Demetrio Ciociaro [1], Veronica Della Latta [1], Anna Paola Pala [1], Annalisa Deodati [3,4], Andrea Raffaelli [1], Filippo Pratesi [1], Raffaele Conte [1], Francesca Maranghi [2], Roberta Tassinari [2], Enrica Fabbrizi [5], Giacomo Toffol [6], Stefano Cianfarani [3,4,7], Cinzia La Rocca [2,*], Amalia Gastaldelli [1,*], LIFE PERSUADED Project Group

Abstract: Phthalates and bisphenol A (BPA) are plasticizers used in many industrial products that can act as endocrine disruptors and lead to metabolic diseases.

During the LIFE PERSUADED project, we measured the urinary concentrations of BPA and Di (2-ethylhexyl) phthalate (DEHP) metabolites in 900 Italian women representative of the Italian female adult population (living in North, Centre and South of Italy in both rural and urban areas).

The whole cohort was exposed to DEHP and BPA with measurable levels above limit of detection in more than 99% and 95% of the samples, respectively. The exposure patterns differed for the two chemicals in the three macro-areas with the highest urinary levels for DEHP in South compared to Central and Northern Italy and for BPA in Northern compared to Central and Southern Italy. BPA levels were higher in women living in urban areas, whereas no difference between areas was observed for DEHP. The estimated daily intake of BPA was 0.11 μg/kg per day, about 36-fold below the current temporary Tolerable Daily Intake of 4 μg/kg per day established by the EFSA in 2015. The analysis of cumulative exposure showed a positive correlation between DEHP and BPA. Further contrasting the exposure to DEHP and BPA, through specific legislative measures, is necessary to limit the harmfulness of these substances.

Supplementary Table S1. DEHP metabolites level in urine samples of mothers residing in the North (N=300), Centre (N=299) or South (N=299) of Italy. In the table are reported the geometric mean (GM) with the 95% CI, the median (P50) and the IQ range (P25-P75). Both urinary (µg/L) and creatinine-adjusted concentrations (µg/g) are shown.

Metabolite	Macro-area	Unit	GM (95% CI)	P50 (P25-P75)	p-value
**MEHP**	**North**[a]	µg/L	4.40 (4.04-4.81)	4.82 (3.14-6.74)	0.0009[a vs b]
	**Centre**[a]		4.73 (4.32-5.19)	4.93 (3.39-7.47)	0.0392[a vs b]
	**South**[b]		4.81 (4.30-5.37)	5.58 (3.90-7.96)	
	**North**	µg/g crea	4.30 (3.91-4.73)	4.41 (2.83-6.58)	
	**Centre**		4.33 (3.88-4.82)	4.16 (2.79-6.79)	
	**South**		4.29 (3.79-4.85)	4.84 (3.15-7.31)	
**MEHHP**	**North**[a]	µg/L	16.2 (14.7-17.8)	16.3 (9.4-29.5)	<0.0001[a vs b]
	**Centre**[a]		16.8 (15.1-18.8)	17.6 (10.0-29.3)	0.0008[a vs b]
	**South**[b]		21.5 (19.4-23.9)	21.7 (12.5-38.6)	
	**North**[a]	µg/g crea	15.6 (14.3-17.0)	15.2 (10.1-25.0)	0.0019[a vs b]
	**Centre**[a]		15.3 (13.9-16.8)	15.1 (9.62-23.2)	0.0005[a vs b]
	**South**[b]		19.0 (17.3-20.9)	18.0 (11.7-30.2)	
**MEOHP**	**North**[a]	µg/L	4.95 (4.51-5.44)	4.99 (2.92-8.50)	<0.0001[a vs b]
	**Centre**[a]		4.81 (4.31-5.36)	5.13 (2.73-8.64)	<0.0001[a vs b]
	**South**[b]		7.09 (6.39-7.87)	6.98 (4.29-12.0)	
	**North**[a]	µg/g crea	4.79 (4.41-5.20)	4.69 (3.10-6.92)	0.0448[a vs b]
	**Centre**[b]		4.39 (4.00-4.81)	4.31 (2.70-6.87)	<0.0001[b vs c]
	Southc		6.33 (5.75-6.96)	6.02 (4.11-9.38)	<0.0001c vs a

a,b,c Different superscript letters indicate statistically significant differences between groups; superscript letters beside the p-values indicate the corresponding pairwise comparison.

Supplementary Table S2. DEHP metabolites level in urine samples of mothers residing in rural (N=448) or in urban (N=450) Italian areas. In the table are reported the geometric mean (GM) with the 95% CI, the median (P50) and the IQ range (P25-P75). Both urinary (µg/L) and creatinine-adjusted concentrations (µg/g) are shown.

Metabolite	Area	Unit	GM (95% CI)	P50 (P25-P75)
**MEHP**	**Rural**[a]	µg/L	4.74 (4.38-5.14)	5.12 (3.52-7.54)
	**Urban**[b]		4.54 (4.20-4.92)	5.04 (3.22-7.21)
	**Rural**	µg/g crea	4.28 (3.91-4.68)	4.49 (2.90-7.20)
	**Urban**		4.33 (3.97-4.73)	4.50 (2.96-6.52)
**MEHHP**	**Rural**	µg/L	17.8 (16.3-19.4)	17.9 (10.5-31.5)
	**Urban**		18.3 (16.8-19.9)	18.1 (10.5-31.7)
	**Rural**	µg/g crea	15.9 (14.7-17.1)	15.6 (10.2-25.0)
	**Urban**		17.2 (16.0-18.6)	16.5 (10.5-27.7)
**MEOHP**	**Rural**	µg/L	5.48 (5.04-5.96)	5.54 (3.25-9.31)
	**Urban**		5.57 (5.12-6.08)	5.66 (3.14-10.1)
	**Rural**[a]	µg/g crea	4.93 (4.58-5.30)	4.81 (3.05-7.60)
	**Urban**[b]		5.29 (4.90-5.71)	5.17 (3.22-8.32)

a,b,c Different superscript letters indicate statistically significant differences between groups; superscript letters beside the p-values indicate the corresponding pairwise comparison.

Supplementary Table S3. DEHP metabolites level in urine samples of mothers (N=898) residing in rural or in urban areas in the three macro-areas. In the table are reported the geometric mean (GM) with the 95% CI, the median (P50) and the IQ range (P25-P75). Both urinary (µg/L) and creatinine-adjusted concentrations (µg/g) are shown.

Macro-Area (MA)	Metabolite	Area	Unit	GM (95% CI)	P50 (P25-P75)	p-value (Area)	p-value (MA)
**North**	**MEHP**	**Rural***	µg/L	7.54 (6.79-8.37)	4.69 (2.92-6.21)		
		**Urban**		7.20 (6.57-7.90)	5.00 (3.30-7.21)		
		**Rural***	µg/g crea	7.13 (6.31-8.05)	4.16 (2.56-6.41)		
		**Urban**		7.17 (6.50-7.90)	4.52 (3.10-6.72)		
	**MEHHP**	**Rural***	µg/L	15.3 (13.1-17.9)	16.4 (9.60-29.8)		
		**Urban***		15.3 (13.2-17.9)	14.9 (8.50-28.3)		
		**Rural***	µg/g crea	14.5 (12.6-16.7)	15.7 (9.46-25.1)		
		**Urban***		15.3 (13.5-17.3)	14.9 (10.2-25.0)		
	**MEOHP**	**Rural***	µg/L	4.81 (4.17-5.55)	4.93 (2.97-8.34)		
		**Urban***		5.00 (4.39-5.69)	5.05 (2.89-8.94)		
		**Rural***	µg/g crea	4.54 (3.99-5.16)	4.71 (3.01-6.69)		0.0310 [N vs C]
		**Urban***		4.97 (4.48-5.52)	4.48 (3.25-7.00)		
**Centre**	**MEHP**	**Rural***	µg/L	8.17 (7.35-9.09)	4.87 (3.56-7.83)		
		**Urban**		7.15 (6.47-7.90)	4.93 (3.15-7.23)		
		**Rural***	µg/g crea	7.23 (6.35-8.23)	4.03 (2.76-7.23)		
		**Urban**		6.87 (6.10-7.74)	4.42 (3.04-6.30)		
	**MEHHP**	**Rural***	µg/L	15.3 (12.6-18.5)	15.9 (9.81-29.6)		
		**Urban***		17.1 (14.9-19.8)	18.6 (10.9-29.3)		
		**Rural[a*]**	µg/g crea	13.3 (11.3-15.7)	13.9 (8.74-21.8)		
		**Urban[b]**		16.3 (14.6-18.2)	16.5 (10.4-25.0)		
	**MEOHP**	**Rural***	µg/L	4.74 (4.06-5.53)	4.87 (2.90-8.19)		
		**Urban***		4.66 (3.88-5.59)	5.48 (2.65-8.92)		
		**Rural***	µg/g crea	4.14 (3.64-4.70)	3.86 (2.62-5.51)		
		**Urban***		4.44 (3.82-5.16)	4.68 (2.74-7.73)		
**South**	**MEHP**	**Rural[a*]**	µg/L	8.77 (7.70-10.00)	5.87 (4.25-8.38)		<0.0001 [S vs N]<br>0.0102 [S vs C]
		**Urban[b]**		7.36 (6.24-8.70)	5.27 (3.11-7.21)	0.0157[b vs a]	
		**Rural[a*]**	µg/g crea	7.74 (6.70-8.93)	5.04 (3.73-8.13)		0.0022 [S vs N]<br>0.0154 [S vs C]
		**Urban[b]**		6.89 (5.80-8.19)	4.49 (2.63-6.39)	0.0301[b vs a]	
	**MEHHP**	**Rural***	µg/L	21.0 (18.4-23.9)	21.5 (13.5-35.8)		0.0043 [S vs N]<br>0.0050 [S vs C]
		**Urban***		20.2 (16.7-24.4)	22.6 (11.6-42.9)		0.0018 [S vs N]<br>0.0261 [S vs C]
		**Rural***	µg/g crea	18.3 (16.4-20.5)	18.2 (13.0-28.3)		0.0192 [S vs N]<br>0.0009 [S vs C]
		**Urban***		18.3 (15.4-21.8)	17.6 (10.8-33.1)		0.0214 [S vs N]
	**MEOHP**	**Rural***	µg/L	7.23 (6.34-8.24)	7.31 (4.35-10.9)		<0.0001 [S vs N]<br><0.0001 [S vs C]
		**Urban***		7.45 (6.44-8.63)	6.63 (4.25-12.4)		0.0004 [S vs N]<br>0.0004 [S vs C]
		**Rural***	µg/g crea	6.34 (5.67-7.08)	5.90 (4.24-9.30)		0.0001 [S vs N]<br><0.0001 [S vs C]
		**Urban**		6.82 (5.98-7.78)	6.06 (4.01-9.70)		0.0011 [S vs N]<br>0.0002 [S vs C]

a,b,c Different superscript letters indicate statistically significant differences between areas; superscript letters beside the p-values (Area) indicate the corresponding pairwise comparison.

* Statistically significant differences between women residing in the same area among the three macro-areas; superscript capital letters (N = North, C =Centre; S = South) beside the p-values (by MA) indicate the corresponding pairwise comparison.

Supplementary Table S4. Relative metabolic rates and percentage fractions of DEHP metabolites in women according to age categories. Data are expressed as medians (IQ range).

AGE		P50 (P25-P75)	p-value
**20-30 (N=19)**	RMR1	4.05 (1.74-7.28)	
	RMR2[a]	3.99 (3.35-4.85)	
	%MEHP	19.8 (12.1-36.5)	
	%MEHHP[a]	55.8 (47.7-65.6)	
	%MEOHP[a]	21.5 (18.5-34.1)	
**30-40 (N=303)**	RMR1[a]	4.94 (3.03-7.78)	0.0164[a vs b]
	RMR2[b]	3.24 (2.35-4.35)	0.0238[b vs a]
	%MEHP[a]	16.8 (11.4-24.8)	0.0164[a vs b]
	%MEHHP	62.0 (54.2-70.2)	
	%MEOHP[b]	19.2 (15.5-24.3)	0.0047[b vs a]
**40-50 (N=333)**	RMR1[a]	4.96 (3.20-7.67)	0.0117[a vs b]
	RMR2[b]	3.10 (2.29-4.11)	0.0107[b vs a]
	%MEHP[a]	16.8 (11.5-23.8)	0.0117[a vs b]
	%MEHHP[b]	62.7 (54.6-70.1)	0.0460[b vs a]
	%MEOHP[b]	19.5 (15.1-23.6)	0.0022[b vs a]
**>50 (N=15)**	RMR1[b]	3.70 (1.81-5.13)	
	RMR2[b]	2.87 (2.42-3.75)	0.0218[b vs a]
	%MEHP[b]	21.3 (16.3-35.6)	
	%MEHHP	58.3 (46.6-66.0)	
	%MEOHP[b]	18.0 (13.8-19.0)	0.0017[c vs a]

a,b,c Different superscript letters indicate statistically significant differences among groups; superscript letters beside the p-values (area) indicate the corresponding pairwise comparison.

Supplementary Table S5. Relative metabolic rates and percentage fractions of DEHP metabolites in women according to BMI categories. Data are expressed as medians (IQ range).

BMI		P50 (P25-P75)	p-value
**<25 (N=672)**	RMR1	4.87 (2.86-7.44)	
	RMR2[a]	3.14 (2.28-4.19)	
	%MEHP	17.0 (11.8-25.9)	
	%MEHHP	62.3 (52.6-70.0)	
	%MEOHP[a]	19.3 (14.9-23.5)	0.0054 [a vs b]
**25-30 (N=150)**	RMR1	5.05 (3.41-7.92)	
	RMR2	3.15 (2.38-4.36)	
	%MEHP	16.5 (11.2-22.7)	
	%MEHHP[a]	64.0 (55.4-69.9)	
	%MEOHP[a]	19.8 (15.9-23.4)	0.0364 [a vs b]

**>30 (N=56)**	RMR1	4.69 (3.21-7.97)	
	RMR2[b]	3.34 (2.59-4.85)	0.0266 [b vs a]
	%MEHP	17.6 (11.2-23.8)	
	%MEHHP[b]	58.9 (53.9-64.3)	0.0423 [b vs a]
	%MEOHP[b]	20.9 (17.5-27.1)	

a,b,c Different superscript letters indicate statistically significant differences among groups; superscript letters beside the p-values (area) indicate the corresponding pairwise comparison.

Supplementary Table S6. Correlations between the sum of DEHP metabolites and BPA levels expressed as unadjusted and adjusted urinary concentrations in women (N=655) residing in different Italian macro-areas and areas. Rho coefficients and p-values are reported.

	**Unadjusted**		**Adjusted**	
	**Rho**	**p-value**	**Rho**	**p-value**
**TOT**	0.1186	**0.0004**	0.1041	**0.0018**
**North**	-0.0260	0.6537	0.0073	0.8998
**Centre**	0.2512	**<0.0001**	0.2561	**<0.0001**
**South**	0.2056	**0.0004**	0.1053	0.0700
**Rural**	0.0750	0.1139	0.1048	**0.0269**
**Urban**	0.1658	**0.0004**	0.1002	**0.0344**

Supplementary Table S7. Correlation between age and the sum of DEHP metabolites or BPA levels as adjusted urinary concentration in women (N=655) living in Italian macro-areas and areas. Rho coefficients and p-values are reported.

		**Σ DEHP Metabolites**		**BPA**	
	N	**Rho**	**p-value**	**Rho**	**p-value**
**TOT**	655	0.0795	**0.0422**	0.0753	0.0541
**North**	254	0.1358	**0.0304**	0.0286	0.6496
**Centre**	193	0.0901	0.2102	0.0895	0.2157
**South**	208	0.0527	0.4497	0.1086	0.1186
**Rural**	319	0.0439	0.4347	0.0519	0.3558
**Urban**	336	0.1029	0.0604	0.0450	0.4110

Supplementary Table S8. Correlation of the sum of DEHP metabolites and BPA as adjusted urinary concentrations with BMI in women (N=879).

		**Σ DEHP Metabolites**		**BPA**	
	**N**	**Rho**	**p-value**	**Rho**	**p-value**
**TOT**	879	0.0217	0.5218	-0.0469	0.1653
**North**	295	-0.0136	0.8159	0.0544	0.3516
**Centre**	290	-0.0808	0.1729	-0.0825	0.1611
**South**	294	0.1180	**0.0442**	-0.0409	0.4860
**Rural**	439	0.0401	0.4028	-0.0575	0.2290
**Urban**	440	0.0063	0.8961	-0.0200	0.6753
